# PhysBinder: improving the prediction of transcription factor binding sites by flexible inclusion of biophysical properties

**DOI:** 10.1093/nar/gkt288

**Published:** 2013-04-24

**Authors:** Stefan Broos, Arne Soete, Bart Hooghe, Raymond Moran, Frans van Roy, Pieter De Bleser

**Affiliations:** ^1^Department for Molecular Biomedical Research, VIB and ^2^Department of Biomedical Molecular Biology, Ghent University, B-9052 Ghent, Belgium

## Abstract

The most important mechanism in the regulation of transcription is the binding of a transcription factor (TF) to a DNA sequence called the TF binding site (TFBS). Most binding sites are short and degenerate, which makes predictions based on their primary sequence alone somewhat unreliable. We present a new web tool that implements a flexible and extensible algorithm for predicting TFBS. The algorithm makes use of both direct (the sequence) and several indirect readout features of protein–DNA complexes (biophysical properties such as bendability or the solvent-excluded surface of the DNA). This algorithm significantly outperforms state-of-the-art approaches for *in silico* identification of TFBS. Users can submit FASTA sequences for analysis in the PhysBinder integrative algorithm and choose from >60 different TF-binding models. The results of this analysis can be used to plan and steer wet-lab experiments. The PhysBinder web tool is freely available at http://bioit.dmbr.ugent.be/physbinder/index.php.

## INTRODUCTION

Proteins called transcription factors (TFs) are crucial for proper regulation of gene expression. They function by binding to regions of DNA called transcription factor binding sites (TFBS). Two different mechanisms contribute to the TF–DNA binding specificity needed for correct regulation of gene expression: a direct readout component caused by direct contact between the amino acids of the protein and the bases of the DNA and an indirect readout component caused by the global shape of the DNA and by conformational changes in both interaction partners (1,2). Traditional methods for predicting TFBS tend to look at the direct readout component alone and almost exclusively at the primary sequence. However, many of these widely used methods, such as positional weight matrices, are afflicted by many false positive predictions, indicating the need for incorporating other discriminative features (3). Recent evidence shows that sequence-dependent structural variations in the DNA account for a significant portion of the protein–DNA specificity (4–6). Thus, it is expected to be beneficial to include structural features and nucleotide dependencies in the prediction models. In a recent publication, we examined the effect of incorporating nucleotide position dependencies, which are related to the 3D structure of the DNA (7), on the prediction of TFBS (8). We also calculated structural features of the DNA and verified to which extent these features improve the prediction of TFBS. We found that incorporation of both types of data can substantially enhance the prediction of TFBS. Here, we present PhysBinder, a web tool based on the flexible Random Forest algorithm published in (8). We compiled >60 vertebrate TF models from various sources, but many more models will be offered in the future, as new data become available. Binding sites for these models can be visualized together with the ENCODE TFBS data track of UCSC genome (9) to get a useful insight in the genomic context of the inspected region.

## INPUT AND OUTPUT

### Input

The PhysBinder web tool is easy to use: for most parameters, we offer default configurations to ensure a quick and easy workflow. Users just provide their sequences of interest and select the appropriate TF model information. Sequences can be uploaded by one of the following means: (i) pasting a set of FASTA-formatted sequences in the input field; (ii) uploading a file with FASTA-formatted sequences; (iii) indicating genomic regions in the ‘Fetch genomic regions’ text field. Subsequently, a model and a threshold are to be selected. We provide three pre-calculated thresholds: ‘Max. Precision’, ‘Max. F-Measure’ and an average of these two measures. A custom threshold can also be selected.

More than 60 different TF models are now available on the PhysBinder website, but we expect to provide more models, as additional data become available. Most of the PhysBinder models are compiled from recent ENCODE data (10), but other sources were also used (see Materials and Methods for more information). TF models constructed from sequences that, according to the literature, clearly contain a sequence element associated with the TF are called ‘direct evidence’ models. When an alternative consensus sequence is found or when no consensus sequence is known for a particular TF, we call the models ‘putative associated factors’ (PAFs). Such a PAF might be a TF binding to multiple sequence elements, or it might be a common cofactor (hence ‘putative associated factor’). By default, PhysBinder is configured to run in filter mode to speed up the calculations. In this mode, sequences are pre-filtered with a short positional weight matrice with low thresholds, minimizing the number of false-negative hits and effectively guaranteeing maximum recall.

### Output

**Figure 1.**
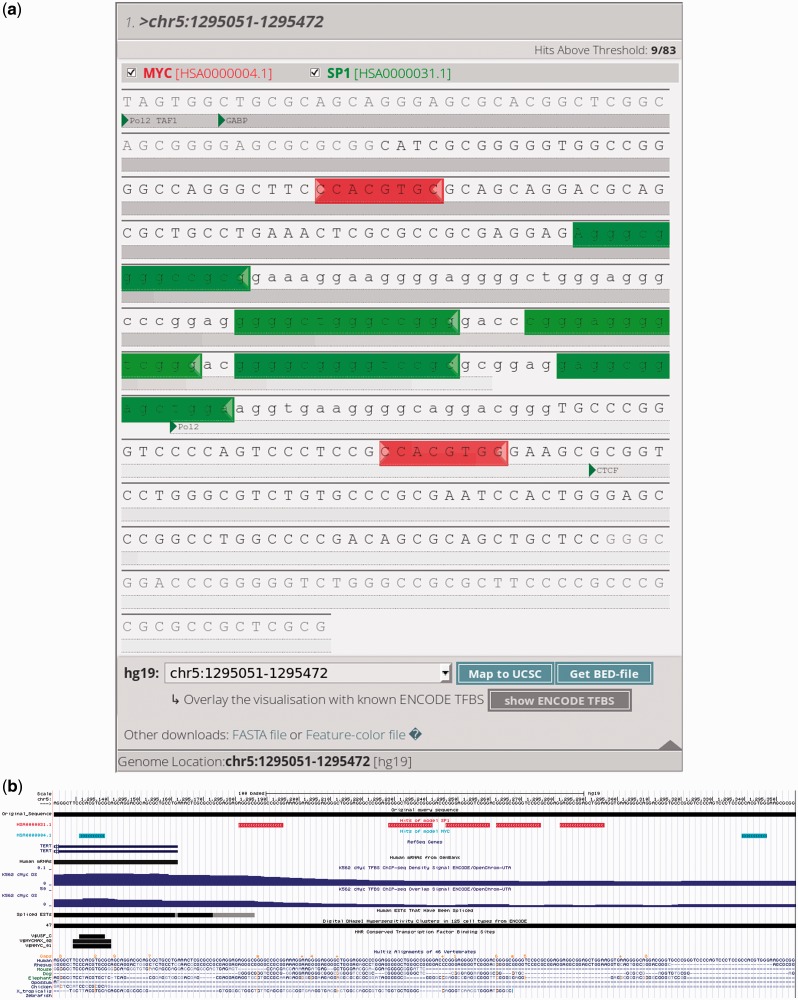
Example output of the PhysBinder tool. All predicted TFBS match the experimentally determined locations reported by Kyo *et al.* (12). (**a**) Detail of the results window: MYC binding sites (E-box) [HSA0000004.1] are shown in red. SP1 binding sites (GC-box) [HSA0000031.1] are shown in green. The default threshold (‘Average’) was used for both models. Gray shaded bars indicate overlapping ENCODE tracks (9). The checkboxes below the sequence indicate the different ENCODE tracks visualized in this sequence. (**b**) Both models were visualized in the UCSC Genome Browser (11). MYC binding sites are indicated in blue, whereas SP1 binding sites are in red.

A summary table is given at the top of the results web page. This table can be sorted by model type or by input sequence, and, for each model or sequence, the number of hits is indicated. On this page, users can still alter the thresholds to increase or decrease the stringency of the binding site predictions. In the results section, binding sites are shown as sequences with a colored background (exemplified in Figure 1a). Clicking on the first nucleotide of such a colored sequence provides more details on the binding site. When clicked, a details window with the sequence logo of the binding site is shown (this logo was calculated on the model data), and the Random Forest score with a *P*-value is given as well. The relative position of the TFBS is shown, and if the genomic location of the sequence is known (because the user indicated this on the input page or performed a BLAT analysis of the sequence against a human or mouse reference genome), then the absolute coordinates of the binding sites are shown in the details window. Two additional options become available when the absolute position is known. For human sequences (hg18 and hg19), it is possible to integrate the most recent ENCODE data to get an overview of the transcription factors and RNA polymerase components that might bind within this genomic region. Predicted binding sites can also be visualized in the UCSC genome browser (11) (exemplified in Figure 1b). Using the aforementioned checkboxes, the sequences or those on the right side of the screen, models can be dynamically shown or hidden to aid the interpretation of the results.

### Example

As an example (see Figure 1), we examined the analysis performed by Kyo *et al.* (12) of the promoter of the human TERT gene, encoding the catalytic subunit of telomerase. These researchers identified a core promoter of 181 bp responsible for the transcriptional activity of the TERT gene. This 181-bp region, consisting of the 5’-UTR and the upstream promoter region, contains two E-boxes bound by MYC *in vivo*. Between these E-boxes, Kyo *et al.* discovered and validated five GC-boxes that are bound by SP1. For illustrative purposes, we used the PhysBinder tool to look for SP1, MYC and TBP binding sites with default threshold settings in the same sequence they used (12), and we were readily able to confirm their findings. We unmistakably found the five SP1 binding sites flanked by two MYC binding sites, as reported in the initial publication. No TATA-box was found, and this promoter was reported to lack such box (13).

## TECHNICAL DETAILS

### Web tool

The web tool is hosted on a Linux CentOS 5 server with 32 GB of RAM, an Apache 2.2.3 web server, and PHP version 5.1.6. Web pages are written in the PHP and Javascript scripting languages. To map input sequences to mouse (mm10) or human (hg19) reference genomes, we use gfServer and Client binaries from UCSC, which makes it possible to BLAT sequences (11). ENCODE tracks are obtained from UCSC Genome (9). Sequences can be fetched from 16 different species, obtained from UCSC Genome. Extensive help documentation is available on the PhysBinder website, including guidelines and tutorials to facilitate the interpretation of the PhysBinder results.

### Backend and models

The backend of PhysBinder is programmed in a combination of Perl and R-script. The Random Forest classifier used in the backend is the ‘FastRandomForest’ implementation. This is a multithreaded implementation of the Random Forest classifier in the Weka statistical package (14). In our models, we use a Random Forest with 100 trees. Most models are built from available ENCODE data of tier 1 cell lines, except for Esrrb (15), ETS1 (16), KLF4 (15), NANOG (15), Nmyc (15), STAT3 (15), TBP (17), Tfcp2l1 (15), TP53 (18) and Zfx (15). All sequences were first aligned using the multiple EM (expectation maximization) for motif elicitation (MEME) motif aligner (19) on the STEVIN supercomputing infrastructure of Ghent University. To ensure the quality of input data, the resulting aligned sequence motifs were then manually searched for in the literature. If a motif is not yet reported in literature, the resulting model is called a PAF. Otherwise, the model is termed a direct evidence model. When available, 100 sequences were used to build the model. The other sequences were used for validation. More information on the different steps of the algorithm and on its validation has been reported by us previously (8). Details about all models are available on the ‘models’ page, where an overview can be found of all the features contained in the models, together with performance measures that were calculated on external test sets.
